# Midgut volvulus with complete malrotation in an infant: First case report from Somalia

**DOI:** 10.1016/j.ijscr.2025.111318

**Published:** 2025-04-20

**Authors:** Mohamed Ahmed Abdillahi, Ahmed Abdi Aw Egge, Kenzu Bedru Hussen, Mumin Farah Ismail, Mohamoud Hashi Abdi, Amina Abdirazak Abadir

**Affiliations:** a Al-Hayatt Hospital, Surgical Department, Borama, Somalia; b College of Health Sciences, School of Medicine and Surgery, Amoud University, Borama, Somalia; c School of Postgraduate Studies and Research, Amoud University, Amoud Valley, Borama, Somalia; d Borama Modern Diagnostic Center, Borama, Somalia

**Keywords:** Midgut volvulus, Intestinal malrotation, Bilious vomiting, Whirlpool sign, Ladd procedure, Case report

## Abstract

**Introduction:**

Malrotation is a congenital anomaly affecting small and large bowels, with 1 in 500 live births globally. It's often accompanied by bilious vomiting, which may occur with or without abdominal distension. Midgut volvulus is a major complication that can arise from malrotation, presenting a significant risk to life and requiring immediate surgical intervention. This is the first case report of midgut volvulus with complete malrotation in an infant from Somalia.

**Case presentation:**

Our case is a 40-day old term male presented with bilious vomiting and constipation for 5 days. He was resuscitated and underwent Color Doppler ultrasound of the abdomen that showed the whirlpool sign and reversal of superior mesenteric artery and superior mesenteric vein. Following the Ladd's procedure, he was discharged from the hospital, demonstrating effective feeding and the absence of vomiting.

**Discussion:**

Intestinal malrotation, a congenital anomaly affecting approximately 1 in 500 live births, carries a significant risk of midgut volvulus, a life-threatening surgical emergency. This report presents the first documented case of complete intestinal malrotation with midgut volvulus in an infant from Somalia, highlighting the challenges of diagnosis and management in resource-constrained settings. The case highlights the importance of accessible diagnostic tools and prompt surgical intervention.

**Conclusion:**

This case highlights the importance of heightened awareness, sonographic expertise, and skilled surgical management of midgut volvulus with complete malrotation to improve outcomes, particularly in underserved regions where access to advanced pediatric care remains limited.

## Highlights

• First Somali Case: Documents the first infant midgut volvulus/malrotation case reported from Somalia.
• Ultrasound Value: Highlights Color Doppler ultrasound (whirlpool sign, SMA-SMV reversal) for rapid diagnosis.
• Resource-Limited Success: Shows successful surgical management is achievable in low-resource settings.
• Positive Surgical Outcome: Notes good results from Ladd's procedure, including quick recovery.
• Advocacy: Calls for better diagnostic/treatment resources in underserved areas.

## 1 Introduction

Malrotation is recognized as a congenital anomaly that involves an abnormal configuration of the intestines within the peritoneal cavity, commonly affecting both the small and large bowel [1]. The incidence of malrotation is around 1 in 500 live births [2]. While specific data for Sub-Saharan Africa is limited, the overall incidence is likely similar to global estimates, but the presentation and management may differ due to factors like access to healthcare and diagnostic tools. In neonates, the typical symptoms of malrotation with midgut volvulus are characterized by bilious vomiting and intestinal obstruction [3]. Case reports about this complication of midgut volvulus in infants are infrequently reported in Sub-Saharan Africa. A thorough review of the literature suggests that this is the first case report documented in the context of Somalia. It highlights the successful provision of supportive care, the clinical diagnostic process, and the surgical treatment carried out, despite the difficulties inherent in a resource-limited setting. This case is composed following the principles outlined in the SCARE 2023 guidelines [4].

## 2 Case presentation

A 40-day-old term male infant presented with bilious vomiting and constipation for 5 days. Previous history was unremarkable. The infant looked sick, severely dehydrated, with moderate abdominal distension, and absent bowel sounds. He weighed 3.4 kg; and his vital signs were as follows: temperature 37.5 °C; pulse rate 165 beats/min; respiratory rate 40 breaths/min; and oxygen saturation of 98 % at room air. Upon transfer to the emergency ward, the dehydration caused by the vomiting was initially corrected, as well as placement of nasogastric tube for bowel decompression, and administration of broad-spectrum antibiotics to cover bowel flora.

**Fig. 1:**
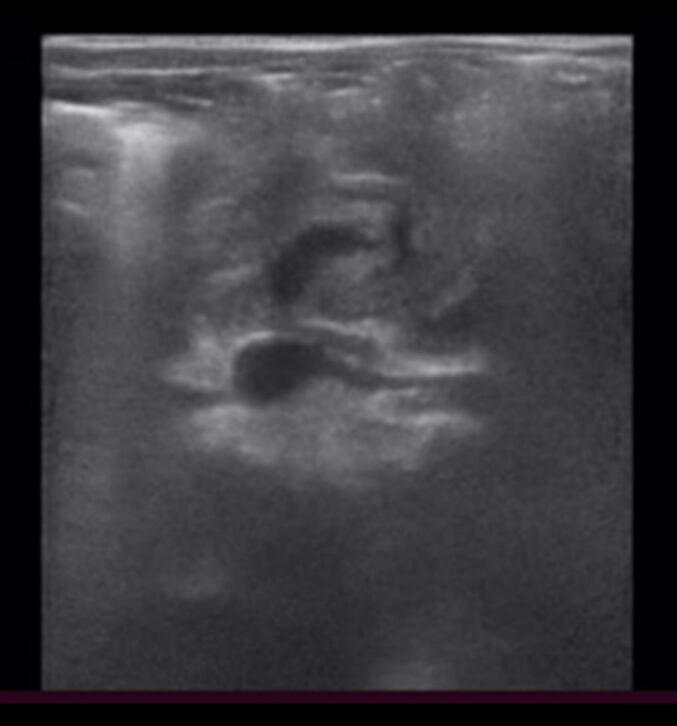
Grey scale ultrasound at the level of the upper abdomen showing whirling vessels and surrounding small bowel loops on transverse scan.

**Fig. 2:**
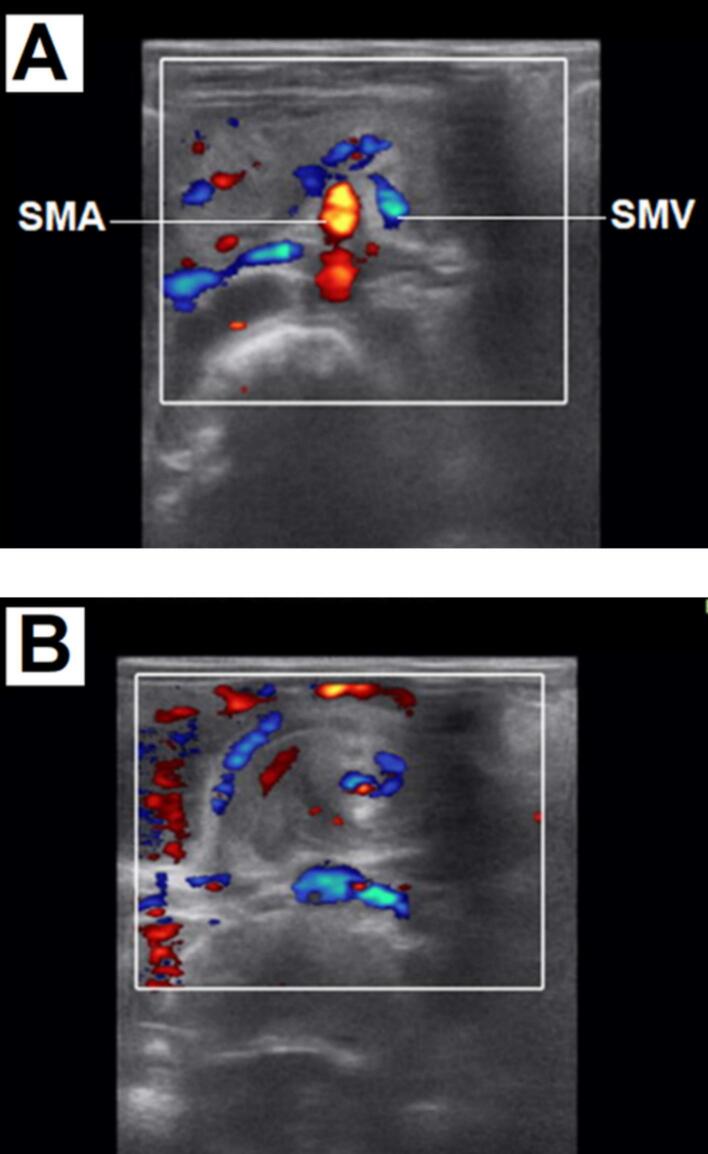
(A) The Color Doppler of upper abdomen shows reversal of SMA-SMV. (B) Color Doppler ultrasound of the upper abdomen shows whirlpool sign.

An abdominal X-ray showed a mild stomach distension and bowel dilation over the left lower quadrant area. Laboratory tests revealed nonspecific findings. A grey-scale ultrasound scanned at the level of the upper abdomen showed whirling vessels and surrounding small bowel loops on transverse scan (Fig. 1). A Color Doppler ultrasound of the upper abdomen showed superior mesenteric artery (SMA) located on the right side and superior mesenteric vein (SMV) on the left side suggesting reversal of SMA-SMV (Fig. 2A). It also showed a whirling of the superior mesenteric vein and small bowel around the superior mesenteric artery on a transverse scan with whirlpool sign (Fig. 2B). Thus the diagnosis of malrotation and midgut volvulus was considered.

**Fig. 3:**
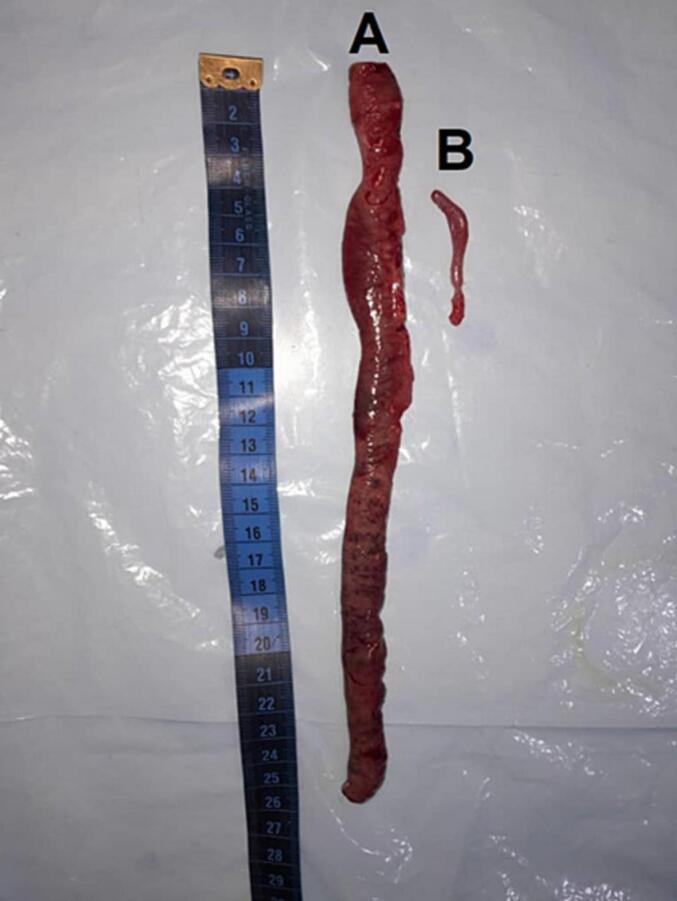
(A) 25 cm of resected proximal jejunum with gangrene. (B) a resected appendix.

**Fig. 4:**
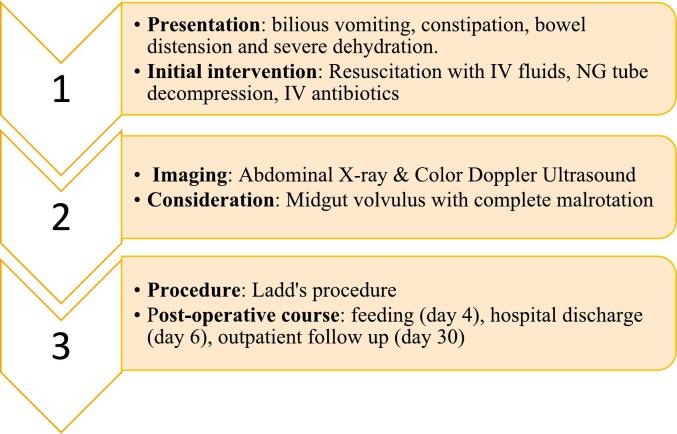
Chronological order from presentation to operation and follow up. (Timeline).

After detailed communication with family members, the infant underwent laparotomy by general surgeons, were a complete malrotation with midgut volvulus is noted. About 25 cm of gangrenous proximal jejunum was found and resected (Fig. 3A), and primary anastomosis was achieved. The midgut volvulus was untwisted after release of multiple Ladd's bands. In addition, an appendectomy was done (Fig. 3B). No persistent vomiting occurred after the operation. The infant was fed on the 4th day after the surgery and was discharged 6 days later. Upon follow-up in the outpatient department one month later, he exhibited no complaints and had reverted to his normal baseline condition (Fig. 4).

## 3 Discussion

Intestinal malrotation is defined as the improper alignment of the bowel loops within the peritoneal cavity that takes place during the intrauterine phase of development [1]. The vast majority of children affected by malrotation do not possess any predisposing syndromes or genetic factors [5].

During the fourth week of embryonic development, the rapidly elongating midgut herniates through the umbilicus because it cannot fit within the abdominal cavity. Around the tenth week, the midgut returns, rotating 270° counterclockwise around the superior mesenteric artery to its normal position. Failure of this rotation causes midgut malrotation, often linked to a fibrous Ladd band that can obstruct the right colon [6].

In a study involving 170 patients of diverse age groups diagnosed with symptomatic intestinal malrotation at a single institution, the age distribution at the time of presentation was as follows: 31 % were infants under one year, 21 % were children aged 1 to 18 years, and 48% were adults over the age of 18 [7].

The leading causes of neonatal intestinal obstruction that result in bilious vomiting are identified as duodenal atresia, jejunoileal atresia, midgut malrotation with associated volvulus, necrotizing enterocolitis, and meconium ileus [8].

Intestinal malrotation typically presents without symptoms, with the majority of diagnoses occurring in childhood, making it a rare condition in adults [9]. A typical clinical presentation of malrotation in infants is characterized by bilious vomiting, which may occur with or without accompanying abdominal distension [1]. During the neonatal stage, the presence of bile-stained vomit is nearly always indicative of an intestinal obstruction [10]. A significant complication associated with this condition is midgut volvulus, which leads to proximal bowel obstruction and ischemia, potentially manifesting as bloody stool in some cases [11]. The majority of neonates experiencing midgut volvulus typically exhibit this complication within the first week following birth, with approximately 80 % of affected individuals developing it during the initial month of life [1]. Malrotation and its associated risk of midgut volvulus pose a complex dilemma for both surgical and radiological professionals [12]. In infants presenting with bilious emesis, acute duodenal obstruction, or abdominal tenderness linked to hemodynamic decline, the diagnosis of intestinal malrotation should be a point of suspicion [13].

The imaging assessment for neonatal bilious vomiting traditionally comprises plain abdominal radiographs and/or contrast studies [8]. The application of ultrasound as a screening technique for malrotation can eliminate the requirement for unnecessary barium studies [14]. Sonography serves as a diagnostic tool for malrotation, highlighting distinctive features such as the reversed orientations of the superior mesenteric artery (SMA) and the superior mesenteric vein (SMV) [15]. The whirlpool sign observed in abdominal sonography of neonates is a widely acknowledged indicator of volvulus resulting from midgut malrotation. This sign manifests as the intestine rotates around its mesentery, with the associated mesenteric vessels and intestinal loops forming a characteristic whirlpool configuration [16]. Moreover, various authors have highlighted the corkscrew sign as a significant diagnostic sign of midgut volvulus [17].

If a patient displays distinct symptoms indicative of intestinal obstruction, this typically points to midgut volvulus, and it is crucial to conduct midgut reduction surgery without delay to relieve the obstruction and restore the normal flow of the intestine. Should intestinal necrosis occur, the procedure will involve intestinal resection and subsequent anastomosis [18]. The management of malrotation with volvulus requires immediate surgical intervention through the Ladd's procedure, regardless of the patient's age or the presence of clinical symptoms. This operation effectively addresses the volvulus, if present, by dividing the Ladd bands, widening the mesenteric base, and repositioning the colon to the left side of the abdomen, with the small intestine situated on the right [19]. Certain surgeons advise against the use of the laparoscopic Ladd's procedure (lap-Ladd) in neonatal patients due to the constraints of the working space. This limitation can hinder the surgeon's ability to accurately locate the positions of all intestinal segments, assess the extent of volvulus, and evaluate whether the expansion of the mesenteric pedicles is adequate [20]. Although appendectomy was not initially included in the Ladd procedure, it is routinely performed in cases where patients are treated surgically for malrotation [21]. The rationale behind performing an appendectomy is generally attributed to two key factors: the positioning of the appendix in the left upper quadrant can result in a delayed or missed diagnosis of acute appendicitis, and the dissection of Ladd's bands could potentially harm the appendiceal vessels that supply blood to the appendix [22]. The incidence of recurrent volvulus following Ladd's procedure is notably low. In some cases, postoperative ileus may necessitate the use of nasogastric tube drainage after laparotomy, with the resumption of full feeding potentially extending over several days, leading to hospital stays of approximately four to five days [23].

The early diagnosis of intestinal malrotation is crucial, as failure to promptly identify and address common complications such as obstruction caused by Ladd's bands or midgut volvulus can lead to life-threatening emergencies [24]. Understanding the likelihood of malrotation in individuals presenting beyond the neonatal stage may contribute to a decrease in diagnostic and treatment delays, as well as a reduction in associated morbidity [25].

## 4 Conclusion

Intestinal Malrotation is congenital that complicates 1 in every 200 births. The principal complication of malrotation is midgut volvulus, which can be a life-threatening condition requiring immediate surgical intervention. The “whirlpool sign” directly indicates the anatomic alteration caused by midgut volvulus. A very high index of suspicion and a solid

understanding of embryology and anatomy is required to promptly diagnose and to appropriately treat malrotation.

This case highlights the importance of recognizing and managing midgut volvulus with complete malrotation, even in environments with limited healthcare resources, where access to pediatric surgical specialists, pediatric intensive care facilities, and diagnostic imaging resources is scarce. It also highlights the favorable surgical outcomes, particularly the quick return to feeding and the relatively short length of hospital stay. Persistent efforts to improve diagnostic capabilities and ensure access to specialized treatment are critical for enhancing the outcomes of patients diagnosed with this rare complication of the intestinal malrotation.

## Patient consent

Informed consent was obtained from the patient's parents for the publication and use of any pertinent images, while guaranteeing the confidentiality of the patient. A copy of this consent document is available for the Editor-in-Chief's review upon request.

## Ethical approval

The ethics committee of our institution granted ethical clearance for this study.

## Guarantor

This paper is under the guarantee of Dr. Mohamed Ahmed Abdillahi.

## Funding

No funding was obtained from any organization.

## Author contribution

1. **Mohamed Ahmed Abdillahi**: Writing the manuscript (drafting the introduction, case description, discussion, and conclusion).
2. **Ahmed Abdi Aw Egge**: Patient data collection (gathering clinical information and relevant medical records).
3. **Kenzu Bedru Hussen**: Critical review and editing (providing feedback and revising the manuscript for clarity and accuracy).
4. **Mumin Farah Ismail**: Critical review and editing (providing feedback and revising the manuscript for clarity and accuracy).
5. **Mohamoud Hashi Abdi**: Conceptualizing the case report (designing the study and identifying key aspects to report).
6. **Amina Abdirazak Abadir**: Data analysis (interpreting findings and drawing conclusions from the case).

## Declaration of competing interest

The authors declare that there are no conflicts of interest related to the publication of this article.
